# Supplementary figures and images for: Delivery mode impacts gut bacteriophage colonization during infancy
Source: medRxiv. 2023 Nov 13:2023.11.13.23298307. Preprint. [Version 1] doi: 10.1101/2023.11.13.23298307 (PMC10680904; doi:10.1101/2023.11.13.23298307)

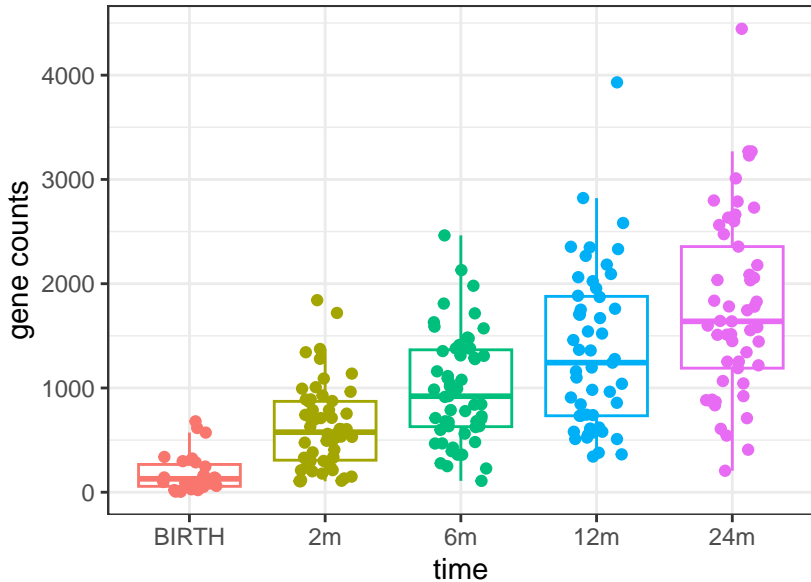

Supplement: Supplement 1 — Figure S1: Identified viral genes over time. [file media-1.pdf]

A

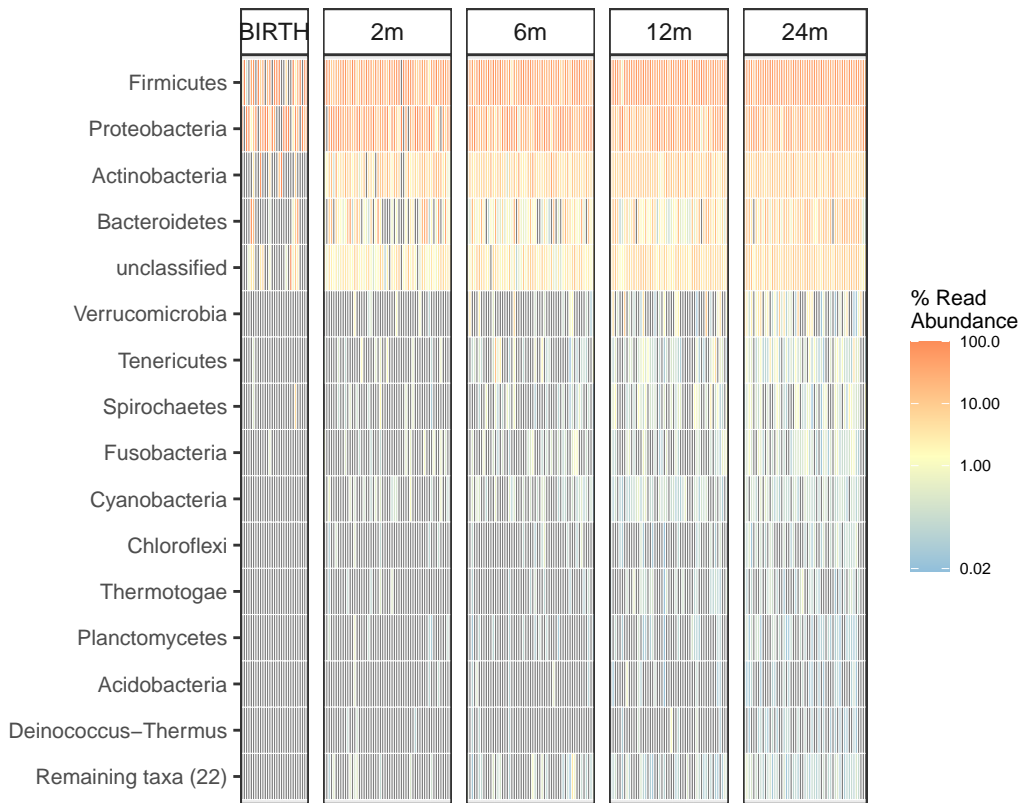

B

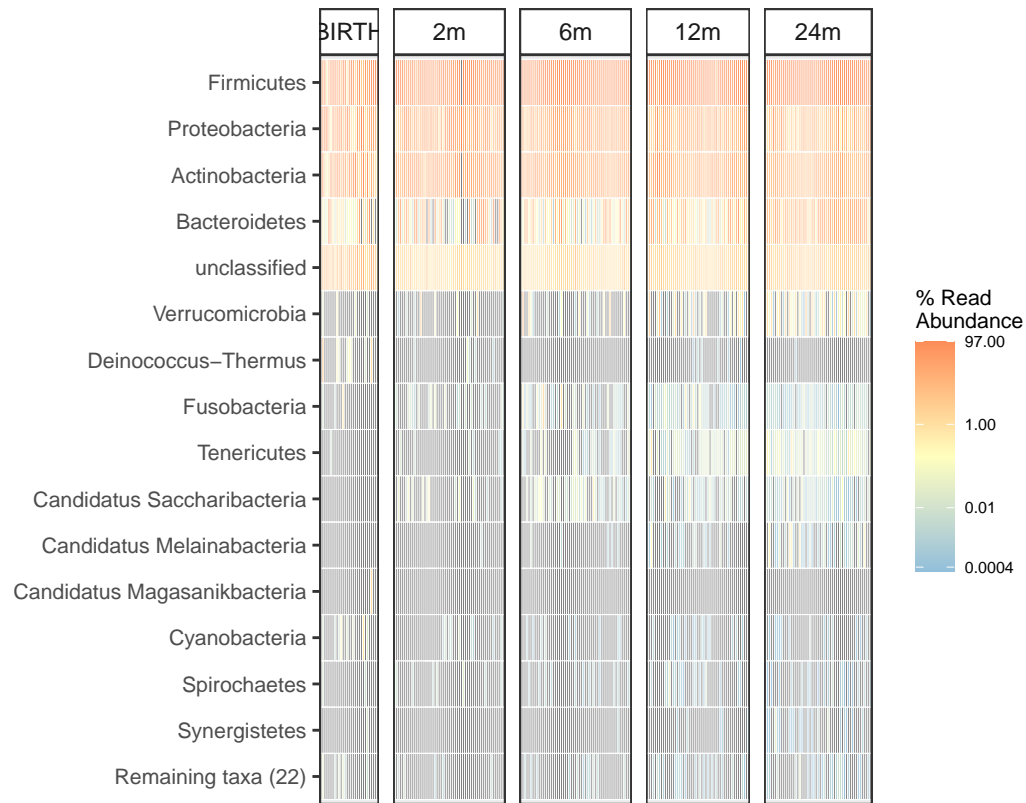

Supplement: Supplement 2 — Figure S2: Predicted host bacterial abundance and whole microbiome bacterial abundance at a phylum level [file media-2.pdf]

A

BIRTH

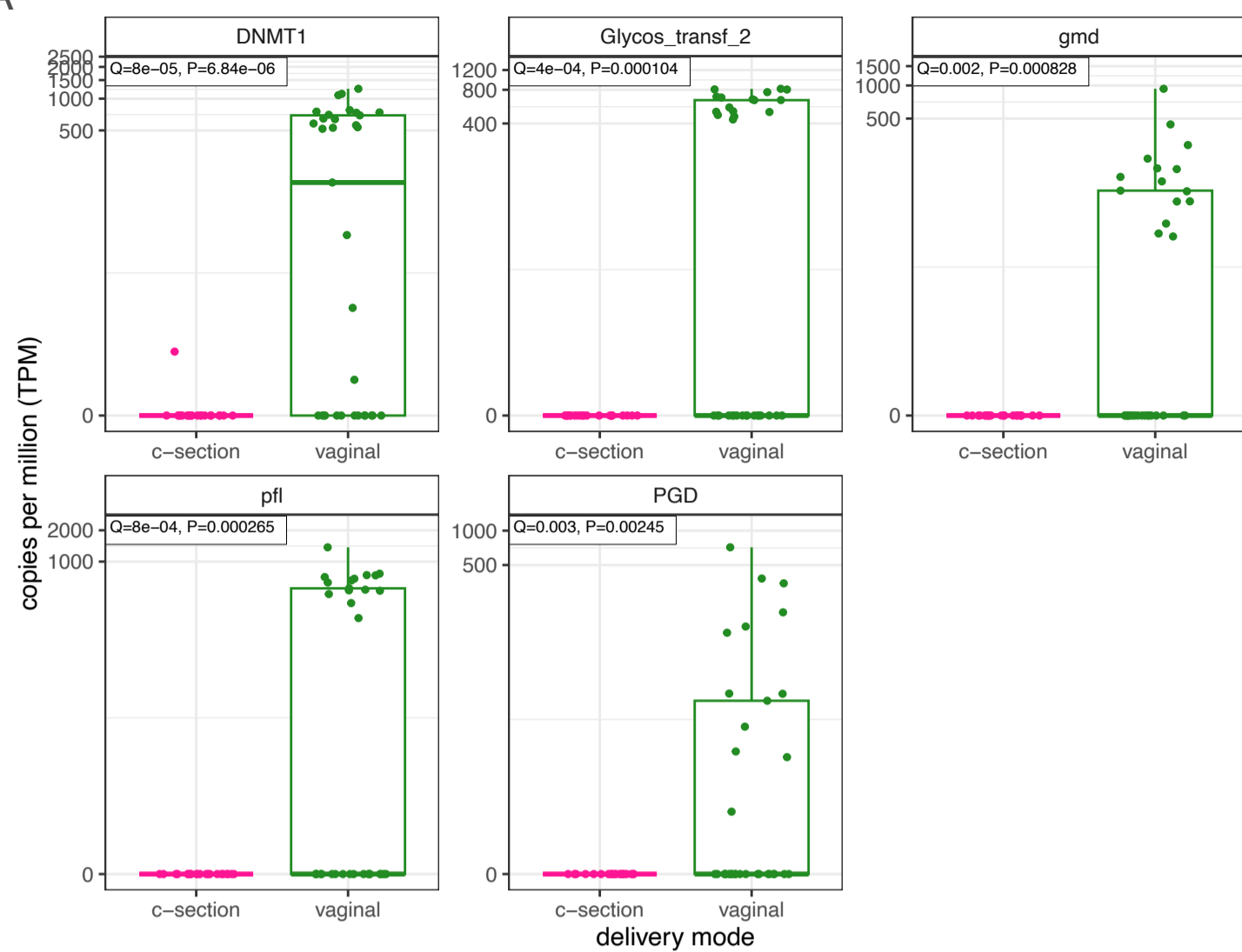

C

6m

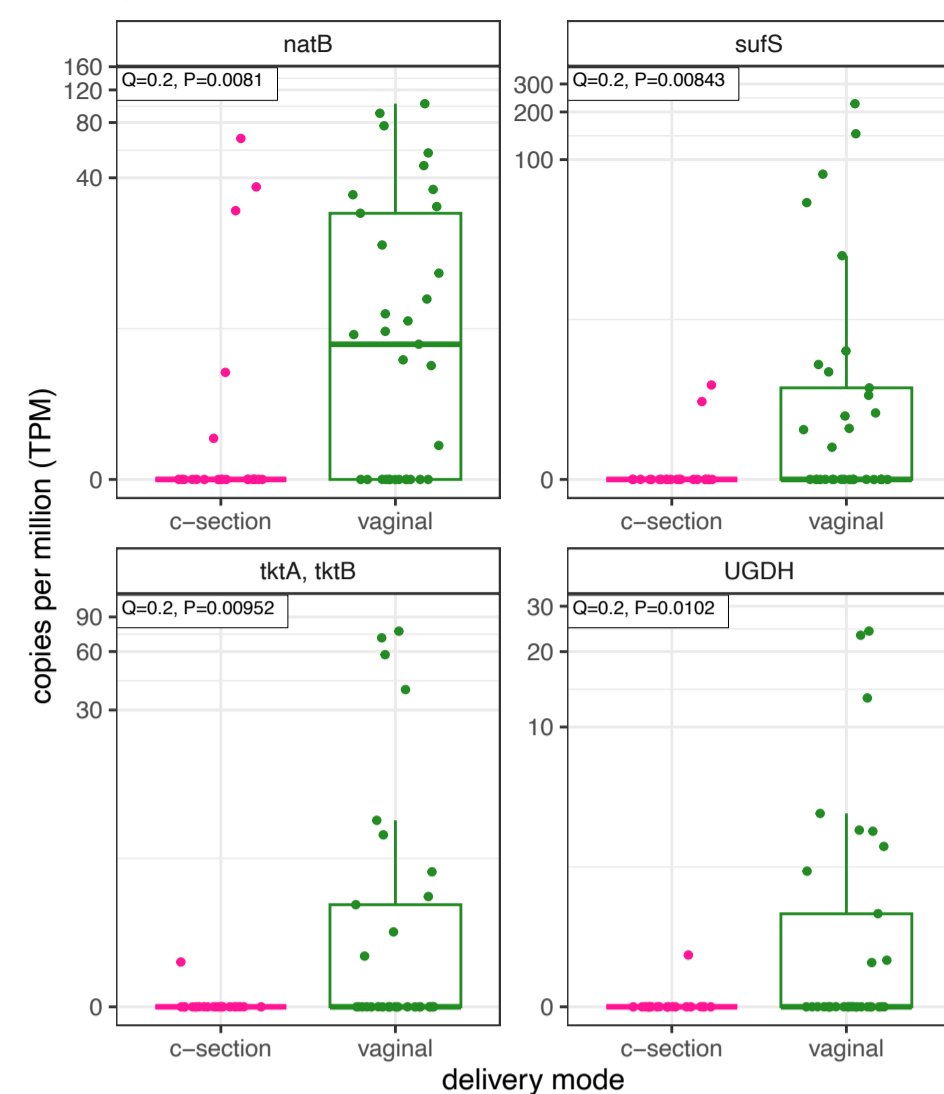

B

2m

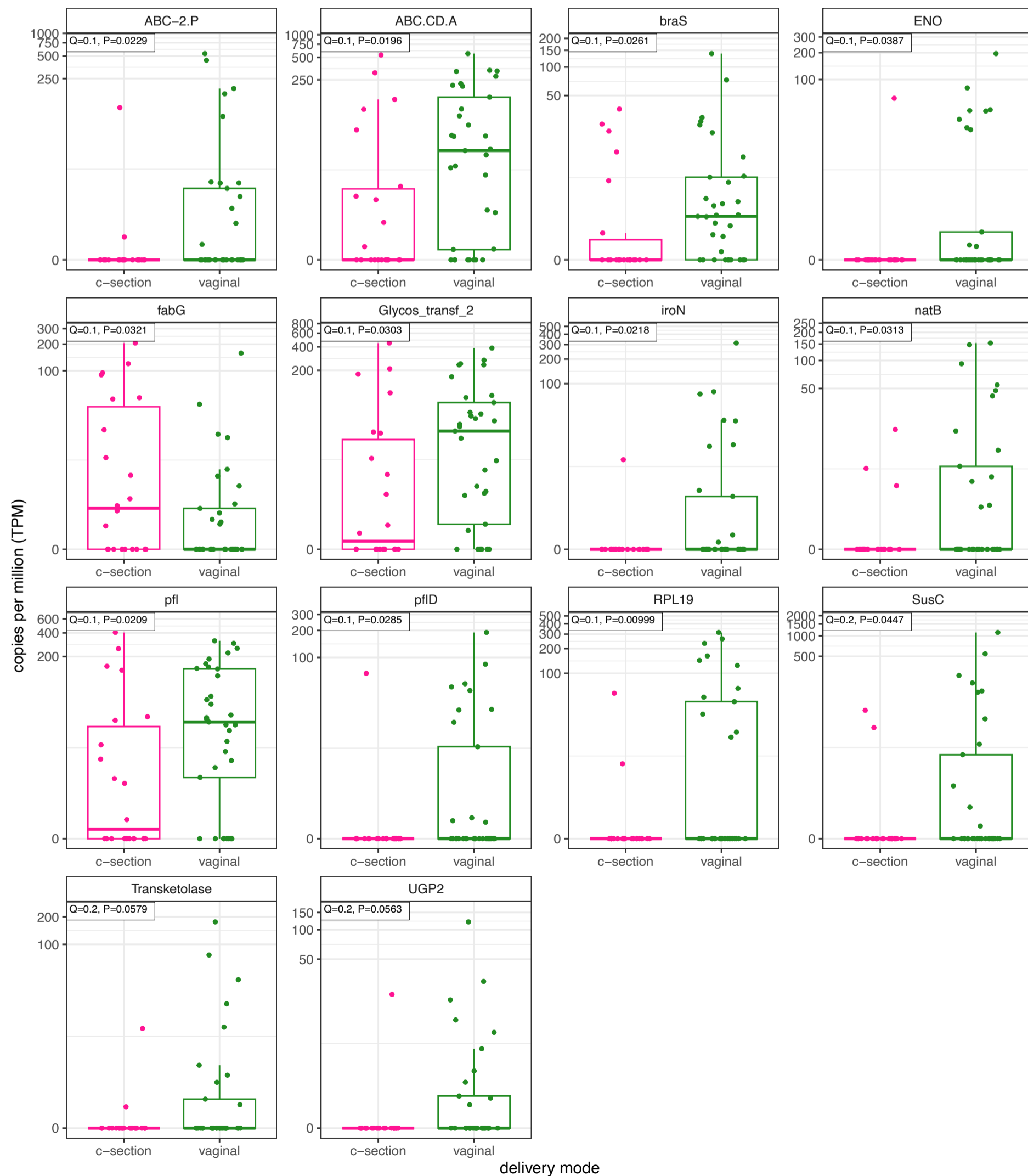

D

24m

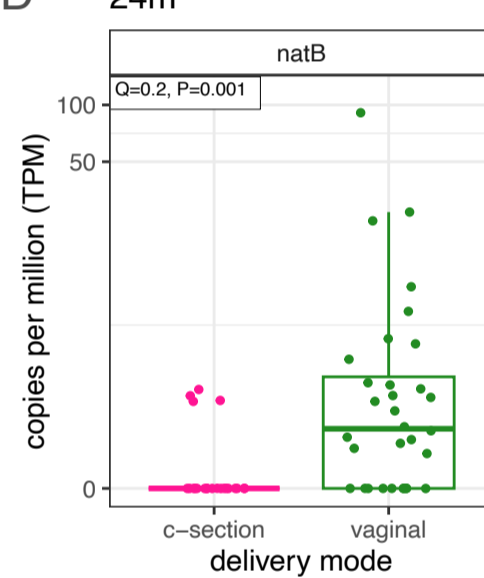

Supplement: Supplement 3 — Figure S3A-D: Differentially abundant viral auxiliary metabolic genes (vAMGs) by delivery mode and timepoint. [file media-3.pdf]
