# Supplementary material for: Delivery mode impacts gut bacteriophage colonization during infancy
Source: medRxiv. 2023 Nov 13:2023.11.13.23298307. Preprint. [Version 1] doi: 10.1101/2023.11.13.23298307 (PMC10680904; doi:10.1101/2023.11.13.23298307)
Supplement: Supplement 4 — Table S1: Demographic and clinical factors stratified by delivery mode. [file media-4.pdf]

Clinical factor	Vaginal delivery (n=33)	Cesarean Section (n=22)	P value
**Sex (n, %)**			
Male	18, 55%	10, 45%	0.509
Female	15, 45%	12, 55%	
**Any Breast feeding at 6 months (n, %)**			
Yes	25, 76%	15, 68%	0.742
No	5, 15%	4, 18%	
Unknown	3, 9%	3, 14%	
**Number of breast feeds per week at 6 months**			
Mean	29.87	31.68	0.167
Range	0 – 99	0 – 70	
**Ethnicity (n, %)**			
Not Hispanic or Latino	31, 94%	21, 95%	1
Hispanic or Latino	2, 6%	1, 5%	
**Race (n, %)**			
White or Caucasian	21, 64%	17, 77%	0.197
Black or African American	5, 15%	1, 4.5%	
Asian	6, 18%	1, 4.5%	
More than one Race	1, 3%	2, 9%	
Other	0, 0%	1, 4.5%	
**Maternal peripartum antibiotics (n, %)**			
Yes	7, 21%	22, 100%	**<0.001**
No	26, 79%	0, 0%	
**Infant antibiotics prior to 2 months (n, %)**			
Yes	3, 9%	4, 18%	0.419
No	30, 91%	18, 82%	
**Infant antibiotics prior to 6 months (n, %)**			
Yes	4, 12%	6, 27%	0.175
No	29, 88 %	16, 73%	
**Infant antibiotics prior to 12 months (n, %)**			
Yes	13, 39%	10, 45%	0.655
No	20, 61%	12, 55%	
**Infant antibiotics prior to 24 months (n, %)**			
Yes	15, 45%	11, 50%	0.741
No	18, 55%	11, 50 %	
