# Supplementary material for: Delivery mode impacts gut bacteriophage colonization during infancy
Source: medRxiv. 2023 Nov 13:2023.11.13.23298307. Preprint. [Version 1] doi: 10.1101/2023.11.13.23298307 (PMC10680904; doi:10.1101/2023.11.13.23298307)
Supplement: Supplement 5 — Table S2: Functions of differentially abundant viral auxiliary metabolic genes (vAMGs) by delivery mode [file media-5.pdf]

Gene	Symbol	Relevant Characteristic(s)	Increased in VD	References
3-oxoacyl-[acyl-carrier-protein] reductase FabG	fabG	Essential for survival in *Escherichia coli*	No (2 m)	1
TonB-dependent starch-binding outer membrane protein SusC	SusC	Essential for utilization of maltooligosaccharides and starch in *Bacteroides thetaiotaomicron*	Yes (2 m)	2
Iron complex outermembrane recepter protein	iroN	Essential for iron uptake in bacterial systems	Yes (2 m)	3
DNA (cytosine-5-)-methyltransferase	DNMT1	Enables DNA methylation targeting and maintenance during cell division	Yes (Birth)	4
UTP--glucose-1-phosphate uridylyltransferase	UGP2	Required to use glucose in anabolic pathways and processes such as glycoprotein folding control, cellular detoxification, and lactation	Yes (2 m)	5
Enolase	ENO	Receptor for human plasminogen that can promote recruitment of monocytes	Yes (2 m)	6,7
Transketolase	tktA, tktB	Required to make erythrose-4-phosphate, which is a precursor of aromatic amino acids and vitamins	Yes (6 m)	8
Glycosyl transferase family 2	Glycos_transf_2	Responsible for galactocerebroside synthesis, which is an antigen that triggers Guillain-Barré-Stohl syndrome	Yes (Birth & 2 m)	9
6-phosphogluconate dehydrogenase	PGD	Important to produce NADPH and could be an anticancer target	Yes (Birth)	10,11
Pyruvate formate lyase activating enzyme	pfI	Activates pyruvate-formate lyase	Yes (Birth & 2 m)	12,13
Formate acetyltransferase	pfID	Catalyzes the nonoxidative generation of formate and acetyl-Coenzyme A	Yes (2 m)	12,13

(pyruvate-formate lyase)				
GDPmannose 4,6-dehydratase	gmd	Catalyzes the transformation of GDP-L-mannose to GDP-L-fucose and is required for exopolysaccharide synthesis in *Caulobacter crescentus*	Yes (Birth)	14,15
Cysteine desulfurase / selenocysteine lyase	sufS	Mobilizes sulfur atoms from cysteine to target proteins during assembly of Fe-S clusters	Yes (6 m)	16
natB; sodium transport system permease protein	natB	Catalyzes ATP-dependent electrogenic Na+ extrusion in absence of mechanistically coupled proton or K+ uptake	Yes (2 m, 6 m & 24 m)	17
braS; two-component system, OmpR family, sensor histidine kinase BraS/BceS	braS	Key regulatory element that allows bacitracin and nisin resistance in *Staphylococcus aureus*	Yes (2 m)	18
ABC-2.P; ABC-2 type transport system permease protein	ABC-2.P	Catalyzes transport of drugs and carbohydrates in bacteria	Yes (2 m)	19
Putative ABC transport system ATP-binding protein	ABC.CD.A	Associated with higher relative abundances in healthy and adenoma samples when compared to cancer samples	Yes (2 m)	20
Large subunit ribosomal protein L19	RPL19	Upregulation induces endoplasmic reticulum stress and cell death in breast cancer cells	Yes (2 m)	21
Transketolase, C-terminal domain	Transketolase	Functions as a regulatory molecule binding site	Yes (2 m)	22
UDP-glucose 6-dehydrogenase	UGDH	Catalyzes the oxidation of UDP-glucose to UDP-glucuronate and could be an anticancer target	Yes (6 m)	23,24

1 Zhang, Y. & Cronan, J. E., Jr. Transcriptional analysis of essential genes of the Escherichia coli fatty acid biosynthesis gene cluster by functional replacement with the analogous Salmonella typhimurium gene cluster. *J Bacteriol* **180**, 3295-3303, doi:10.1128/jb.180.13.3295-3303.1998 (1998).

2 Reeves, A. R., D'Elia, J. N., Frias, J. & Salyers, A. A. A Bacteroides thetaiotaomicron outer membrane protein that is essential for utilization of maltooligosaccharides and starch. *J Bacteriol* **178**, 823-830, doi:10.1128/jb.178.3.823-830.1996 (1996).

3 Clarke, T. E., Tari, L. W. & Vogel, H. J. Structural biology of bacterial iron uptake systems. *Curr Top Med Chem* **1**, 7-30, doi:10.2174/1568026013395623 (2001).

4 Zhang, G. *et al.* Small RNA-mediated DNA (cytosine-5) methyltransferase 1 inhibition leads to aberrant DNA methylation. *Nucleic Acids Res* **43**, 6112-6124, doi:10.1093/nar/gkv518 (2015).

5 Führing, J. I. *et al.* A quaternary mechanism enables the complex biological functions of octameric human UDP-glucose pyrophosphorylase, a key enzyme in cell metabolism. *Sci Rep* **5**, 9618, doi:10.1038/srep09618 (2015).

6 Candela, M. *et al.* Bifidobacterial enolase, a cell surface receptor for human plasminogen involved in the interaction with the host. *Microbiology (Reading)* **155**, 3294-3303, doi:10.1099/mic.0.028795-0 (2009).

7 Wygrecka, M. *et al.* Enolase-1 promotes plasminogen-mediated recruitment of monocytes to the acutely inflamed lung. *Blood* **113**, 5588-5598, doi:10.1182/blood-2008-08-170837 (2009).

8 Harinarayanan, R., Murphy, H. & Cashel, M. Synthetic growth phenotypes of Escherichia coli lacking ppGpp and transketolase A (tktA) are due to ppGpp-mediated transcriptional regulation of tktB. *Mol Microbiol* **69**, 882-894, doi:10.1111/j.1365-2958.2008.06317.x (2008).

9 Gaspari, E., Koehorst, J. J., Frey, J., Martins Dos Santos, V. A. P. & Suarez-Diez, M. Galactocerebroside biosynthesis pathways of Mycoplasma species: an antigen triggering Guillain-Barré-Stohl syndrome. *Microb Biotechnol* **14**, 1201-1211, doi:10.1111/1751-7915.13794 (2021).

10 Hanau, S. & Helliwell, J. R. 6-Phosphogluconate dehydrogenase and its crystal structures. *Acta Crystallogr F Struct Biol Commun* **78**, 96-112, doi:10.1107/s2053230x22001091 (2022).

11 Lin, R. *et al.* 6-Phosphogluconate dehydrogenase links oxidative PPP, lipogenesis and tumour growth by inhibiting LKB1-AMPK signalling. *Nat Cell Biol* **17**, 1484-1496, doi:10.1038/ncb3255 (2015).

12 Stairs, C. W., Roger, A. J. & Hampl, V. Eukaryotic pyruvate formate lyase and its activating enzyme were acquired laterally from a Firmicute. *Mol Biol Evol* **28**, 2087-2099, doi:10.1093/molbev/msr032 (2011).

13 Zelcbuch, L. *et al.* Pyruvate Formate-Lyase Enables Efficient Growth of Escherichia coli on Acetate and Formate. *Biochemistry* **55**, 2423-2426, doi:10.1021/acs.biochem.6b00184 (2016).

14 Jang, M.-H. *et al.* Molecular cloning of the genes for GDP-mannose 4, 6-dehydratase and GDP-l-fucose synthetase from Bacteroides thetaiotaomicron. *Food Science and Biotechnology* **19**, 849-855, doi:10.1007/s10068-010-0120-0 (2010).

15 Herr, K. L. *et al.* Exopolysaccharide production in Caulobacter crescentus: A resource allocation trade-off between protection and proliferation. *PLoS One* **13**, e0190371, doi:10.1371/journal.pone.0190371 (2018).

16 Loiseau, L., Ollagnier-de-Choudens, S., Nachin, L., Fontecave, M. & Barras, F. Biogenesis of Fe-S cluster by the bacterial Suf system: SufS and SufE form a new type of cysteine desulfurase. *J Biol Chem* **278**, 38352-38359, doi:10.1074/jbc.M305953200 (2003).

17 Cheng, J., Guffanti, A. A. & Krulwich, T. A. A two-gene ABC-type transport system that extrudes Na+ in Bacillus subtilis is induced by ethanol or protonophore. *Mol Microbiol* **23**, 1107-1120, doi:10.1046/j.1365-2958.1997.2951656.x (1997).

18 Hiron, A., Falord, M., Valle, J., Débarbouillé, M. & Msadek, T. Bacitracin and nisin resistance in Staphylococcus aureus: a novel pathway involving the BraS/BraR two-component system (SA2417/SA2418) and both the BraD/BraE and VraD/VraE ABC transporters. *Mol Microbiol* **81**, 602-622, doi:10.1111/j.1365-2958.2011.07735.x (2011).

19 Reizer, J., Reizer, A. & Saier, M. H., Jr. A new subfamily of bacterial ABC-type transport systems catalyzing export of drugs and carbohydrates. *Protein Sci* **1**, 1326-1332, doi:10.1002/pro.5560011012 (1992).

20 Ai, D., Pan, H., Li, X., Wu, M. & Xia, L. C. Association network analysis identifies enzymatic components of gut microbiota that significantly differ between colorectal cancer patients and healthy controls. *PeerJ* **7**, e7315, doi:10.7717/peerj.7315 (2019).

21 Hong, M., Kim, H. & Kim, I. Ribosomal protein L19 overexpression activates the unfolded protein response and sensitizes MCF7 breast cancer cells to endoplasmic reticulum stress-induced cell death. *Biochem Biophys Res Commun* **450**, 673-678, doi:10.1016/j.bbrc.2014.06.036 (2014).

22 Lee, H., Deng, M., Sun, F. & Chen, T. An integrated approach to the prediction of domain-domain interactions. *BMC Bioinformatics* **7**, 269, doi:10.1186/1471-2105-7-269 (2006).

23 Arnold, J. M. *et al.* UDP-glucose 6-dehydrogenase regulates hyaluronic acid production and promotes breast cancer progression. *Oncogene* **39**, 3089-3101, doi:10.1038/s41388-019-0885-4 (2020).

24 Zimmer, B. M., Barycki, J. J. & Simpson, M. A. Integration of Sugar Metabolism and Proteoglycan Synthesis by UDP-glucose Dehydrogenase. *J Histochem Cytochem* **69**, 13-23, doi:10.1369/0022155420947500 (2021).
